# Validation of an Arabic version of the eating disorder inventory’s body dissatisfaction subscale among adolescents, adults, and pregnant women

**DOI:** 10.1186/s40337-023-00911-y

**Published:** 2023-10-19

**Authors:** Sarah Gerges, Sahar Obeid, Diana Malaeb, Abir Sarray El Dine, Rabih Hallit, Michel Soufia, Feten Fekih-Romdhane, Souheil Hallit

**Affiliations:** 1 School of Medicine and Medical Sciences, Holy Spirit University of Kaslik, P.O. Box 446, Jounieh, Lebanon; 2 Department of Social and Education Sciences, School of Arts and Sciences, Lebanese American University, Byblos, Lebanon; 3 College of Pharmacy, Gulf Medical University, Ajman, United Arab Emirates; 4 Department of Biomedical Sciences, School of Arts and Sciences, Lebanese International University, Beirut, Lebanon; 5 Department of Infectious Disease, Bellevue Medical Center, Mansourieh, Lebanon; 6 Department of Infectious Disease, Notre Dame Des Secours, University Hospital Center, Byblos, Lebanon; 7 The Tunisian Center of Early Intervention in Psychosis, Department of Psychiatry “Ibn Omrane”, Razi Hospital, 2010 Manouba, Tunisia; 8 Faculty of Medicine of Tunis, Tunis El Manar University, Tunis, Tunisia; 9 Applied Science Research Center, Applied Science Private University, Amman, 11931 Jordan; 10 Research Department, Psychiatric Hospital of the Cross, P.O. Box 60096, Jal Eddib, Lebanon

**Keywords:** Body image, Body dissatisfaction, Eating disorders, Pregnancy, Adolescents, Adults, Psychometric properties, Cultural adaptation

## Abstract

**Introduction:**

The 9-item Body Dissatisfaction Subscale (BDS) of the Eating Disorder Inventory is one of the most used tools for assessing thinness-oriented body dissatisfaction in research and clinical practice. However, no validated Arabic version of this scale exists to date. In this study, we sought to validate this instrument in three samples of native Arabic-speaking adolescents, adults, and pregnant women from Lebanon.

**Methods:**

A total of 826 adults, 555 adolescents, and 433 pregnant women were included. To examine the factor structure of the BDS, we performed an exploratory factor analysis (EFA), using a principal component analysis via the FACTOR software on the first split-half subsample among Lebanese adults. We used data from the second split-half in the adult sample to conduct a Confirmatory Factor Analysis (CFA) through the SPSS AMOS v.29 software. That verified model was tested via CFA on adolescents and pregnant women.

**Results:**

The EFA showed a bidimensional structure for the BDS, with all 9 items retained and divided into Factor 1 = Body Satisfaction (negatively-worded items) and Factor 2 = Body Dissatisfaction (positively-worded items). The CFA demonstrated invariable goodness-of-fit of the instrument in the three studied populations. McDonald’s omega values were also adequate in the three samples, demonstrating its reliability. Moreover, the BDS showed invariance across sex among both adolescents and adults. Finally, higher BDS scores were correlated with more disordered eating, less body appreciation and less functionality appreciation, thus attesting to convergent validity of the scale. In addition, BDS scores correlated positively with depression and anxiety scores, indicating adequate patterns of divergent validity.

**Conclusion:**

In light of our findings, we endorse the use of the BDS by healthcare professionals in Arabic-speaking countries, in order to assess thinness-oriented body dissatisfaction in an appropriate and timely manner and ease early referral to a specialist, thereby preventing the deleterious health-related risks associated with this condition.

**Supplementary Information:**

The online version contains supplementary material available at 10.1186/s40337-023-00911-y.

## Abstract (plain language summary)

Body dissatisfaction is conceptualized as the pejorative subjective assessment of one's own body and discontent with the visual perception of it or with a particular feature of appearance, which is considered to be the most important global measure of stress related to the body. Body dissatisfaction has been found to be a predictor for the development of an eating disorder and occurs in individuals with different mental disorders. The 9-item Body Dissatisfaction Subscale (BDS) of the Eating Disorder Inventory is one of the most used tools for assessing thinness-focused body dissatisfaction, but has not yet been validated in Arabic. To this end, we aimed to translate and validate the Arabic version of the BDS in the present study, which would in turn facilitate research and improve clinical practices related to body dissatisfaction in Arabic-speaking nations. The present findings provide support for the psychometric properties of the Arabic version of the BDS for examining body dissatisfaction in Arabic-speaking adolescents, adults, and pregnant women in Lebanon.

**Supplementary Information:**

The online version contains supplementary material available at 10.1186/s40337-023-00911-y.

## Introduction

Within recent decades, research into body dissatisfaction and its adverse outcomes has significantly risen [1–3]. In contrast to common belief, body image dissatisfaction constitutes a widespread concern among the general population of both sexes and different age ranges, and does not limit to young adult women [4–11]. As a result, researchers have generated a wide variety of tools for assessing this construct, including measures of both thinness-oriented and muscularity-oriented body dissatisfaction as well as measures of positive aspects of body image [12].

An international and widely employed tool for assessing thinness-oriented body dissatisfaction in research and clinical practice worldwide is the 9-item Body Dissatisfaction Subscale (BDS) of the Eating Disorder Inventory [13–16], a highly internally consistent self-report scale developed by Garner et al. [17]. Researchers confirmed the EDI’s psychometric adequacy (i.e., validity and reliability) in a variety of countries, populations, and age groups [18]; including Swedish [19, 20], Chinese [21], Taiwanese [22], Korean [23], Austrian [24], Chilean [25], and Japanese adults [26] as well as American [27], Chilean [25], German [28], Argentinian [29], and French adolescents [30]. In particular, the 9 items of the BDS demonstrated consistent and excellent psychometric performance and reliability across languages and populations, remaining unchanged between the original version (EDI) and subsequent revision (EDI-2) [17, 31]. Regarding the validation of the EDI in the Arabic language, it is noteworthy that the original validation had to exclude measures of the body satisfaction subscale, underscoring the importance of investigating the body dissatisfaction construct via the BDS in the Lebanese cultural and linguistic context [32].

### Body dissatisfaction during the unique pregnancy period

Another population which has consistently shown an increased susceptibility to body image dissatisfaction is pregnant women [33–36], as pregnancy results in significant physical changes and increased societal pressures related to appearance, making it an emotionally critical period of life [37]. Body dissatisfaction experienced during the vulnerable pregnancy period frequently engenders pregnancy-related anxiety, depressive symptoms, weight gain stigma, and disordered eating attitudes [38–41]. Remarkably, Lebanese pregnant women were found to experience notable levels of depression, anxiety, and disordered eating attitudes [42–45]. As a result, validated easy-to-use screening instruments for body dissatisfaction during pregnancy are also highly needed in the Lebanese population since early diagnosis of a condition is one of the key factors for a positive prognosis [46, 47].

The psychometric performance and applicability of the EDI were investigated in the context of pregnancy in only one study in Hungary [48], revealing a strong internal consistency for the BDS. However, the analysis necessitated the removal of one item, specifically "I think that my stomach is too big," resulting in the adaptation of eight statements for pregnant women [48]. This modification was justified by the fact that weight gain in anterior body parts is inherent to pregnancy, and a large stomach can therefore be considered normal and acceptable.

### Rationale and objectives of the current study

Despite the presence of Arabic validated measures of muscularity-oriented body dissatisfaction (i.e., the Arabic Muscle Dysmorphic Disorder Inventory [49]) and positive body image (i.e., the Arabic body appreciation scale-2 [50]), no prior study has evaluated the suitability and measurement properties of an instrument specific to thinness-oriented body dissatisfaction, a frequent negative aspect of body image. Thinness-oriented body dissatisfaction is a critical construct to examine within cultural contexts, as it reflects the subjective experience of individuals and their perceptions of their own bodies [51], which can be influenced by various socio-cultural factors. For instance, research discovered that as Arab countries modernized and Westernized, younger generations became increasingly fixated with Western ideal standards of body size and shape [52, 53]. Given the unique cultural values, norms, and beauty ideals prevalent in the Lebanese society [54], it is crucial to explore the manifestation and impact of body dissatisfaction among individuals, as this can contribute to a better understanding of the etiology and maintenance of eating disorder symptoms [55–57], as well as inform culturally sensitive interventions. Although an Arabic translation of the BDS has been used in previous research [58–60], its psychometric properties have not yet been investigated.

Taking all of preceding into account, our study’s objective was to psychometrically validate an Arabic-translated version of the BDS in three different subgroups (i.e., adolescents, adults, and pregnant women). The body dissatisfaction subscale of the EDI was chosen for its widespread use, comparability across studies, comprehensive assessment of thinness-oriented body dissatisfaction dimensions, and established reliability and validity across diverse cultural contexts [13–16, 18]. We specifically aimed to examine its psychometric performance (i.e., factor structure, reliability, and convergent/divergent validity), as well as its psychometric equivalence (i.e., measurement invariance) between sexes. Namely, divergent validity was assessed using measures of depression and anxiety [57, 61]. In accordance with previous literature showing inverse correlations between body/functionality appreciation and body dissatisfaction [62–64], as well as positive associations between body dissatisfaction and eating disorder symptoms [55, 56], measures of eating disorder symptoms, functionality appreciation, and body appreciation were used for establishing convergent validity. Finally, given the well-known cultural differences in pregnancy-related eating behaviors, dietary patterns, and body image concerns [65–68], we decided to start from the total pool of items and to test the relevance of the item “thinking one’s stomach is too big” in the Arab pregnant women population.

## Methods

### Study 1: adolescents

#### Study design and procedures

Between May and June 2020, we conducted a cross-sectional study among Lebanese adolescents. All the information was gathered through the snowball sampling technique by using a Google Form link shared on social media platforms (WhatsApp and Facebook). A copy was sent to their parents in order to obtain parental consent on their participation. A projected completion duration for the questionnaire was included in the project’s social media advertising. Being a resident and citizen of Lebanon was a requirement for participation. The questionnaire’s instruments were given to participants in a pre-randomized order to account for order effects after their parents had given digital informed consent. Participants in the survey responded anonymously, voluntarily, and without payment. At the conclusion of the survey, debriefing materials were distributed to each participant. The participants and their parents were also asked to send the questionnaire to other participants they knew. In total, 555 adolescents were reached.

#### Questionnaires and measures

The distributed questionnaire was divided into two sections. In the first section, participants were asked to provide demographic information such as their sex and age. Self-reported BMI as kg/m2 was calculated using height and weight data. The household crowding index (HCI), reflecting the socioeconomic status (SES), was calculated by dividing the number of persons by the number of rooms in the house; higher HCI scores reflect lower SES [69].

The second part included the following scales:

*The Body Dissatisfaction Scale (BDS) of the Eating Disorder Inventory* [17] (for which we report the psychometric properties in this paper): It is a part of the lengthy EDI that evaluates the psychology of eating is this 9-item subscale. The BDS items are used to assess thinness-oriented body dissatisfaction (e.g., “I think my buttocks are too large”), which are scored from 0 (“sometimes/rarely/never”) to 3 (“always”). Five questions are negatively-worded (e.g., “I feel satisfied with the shape of my body”). The total score falls between 0 and 27, and is calculated after reversing the score of negatively-worded items. The higher the sum of items, the higher the body dissatisfaction [17]. The Arabic version was used in a previous paper [58] and can be found as a Additional file 1.

*The Patient Health Questionnaire (PHQ-9):* This short 9-item tool is highly efficacious in depicting depressive disorders [70]. Each item (e.g., “Little interest or pleasure in doing things” and “Feeling down, depressed, or hopeless”) is scored from 0 (“not at all”) to 3 (“nearly every day”), in order to quantify the severity of symptoms [70]. This unidimensional structure of the scale was also been validated in Arabic among the Lebanese population [71] (α = 0.84).

*The Hamilton Anxiety Rating Scale (HAM-A):* The HAM-A, a 14-item 5-point Likert instrument, appraised the presence and severity of anxious symptoms (scores between 0 and 56) [72]. Items include “Difficulty in falling asleep, broken sleep, unsatisfying sleep and fatigue on waking, dreams, nightmares, night terrors.”. Lately, this scale was validated in Lebanon [73]. Higher scores indicate higher level of anxiety. (α = 0.89).

*The Restraint Scale of the Dutch Eating Behavior Questionnaire (DEBQ-R):* It is a brief scale that assess the frequency of dieting (e.g., “When you have put on weight, do you eat less than you usually do?” and “Do you try to eat less at meal times than you would like to eat?”). Ten items are rated on a 5-point Likert scale, where response options range between 1 (“never”) to 5 (“always”). Higher scores indicate greater dietary restraint [74]. This scale has also been validated in the Arabic language in Lebanon, with all items loading on one factor [75, 76] (α = 0.92).

*The Düsseldorf Orthorexia Scale (DOS):* This tool includes ten items measuring orthorexia nervosa tendencies (e.g., feeling distressed after eating unhealthy food, following rules to maintain a healthy diet, etc.), answered on a four-point Likert scale ranging from 1 (“never”) to 4 (“always”) [77]. It was validated in the Arabic language in Lebanon as a one-factor structure [78] (α = 0.85).

### Study 2: adults

#### Study design and procedures

Data collection occurred between December 2021 and April 2022 through a Google Form link. The project was promoted on social media platforms (WhatsApp and Facebook) through the snowball sampling technique, indicating an estimated timeframe for completion. To be eligible for participation, individuals had to be adult residents and citizens of Lebanon. To ensure data integrity, IP addresses were examined to prevent duplicate survey submissions. Upon providing digital informed consent, participants were instructed to complete the aforementioned instruments presented in a randomized order to mitigate any potential order effects. The survey maintained anonymity, and participants voluntarily completed it without receiving any form of compensation. In total, 826 adults were reached.

#### Questionnaires and measures

The distributed questionnaire was divided into two sections. The first section was the same as study 1. The second section included the following scales:

*The Body Dissatisfaction Scale (BDS) of the Eating Disorder Inventory* [17]: The description is given in study 1.

*The Body Appreciation Scale-2 (BAS-2):* This test is composed of 10 items measuring acceptance, respect, and care for one's body, as well as its protection from unattainable beauty standards (e.g., "I respect my body"). The total score is determined by averaging the ratings given for each item, which range from 1 (“never”) to 5 (“always”). Greater body appreciation is reflected in higher scores on this scale [79]. This unidimensional model of the scale is validated in Arabic [50] (α = 0.96).

*The Functionality Appreciation Scale (FAS):* The FAS is a short 7-item scale assessing the functionality appreciation of one’s own body: a conceptualized construct that goes beyond simple awareness of the body's capabilities to include respect, honor, and appreciation of what it is capable of doing. Items assess a variety of bodily functions, without restriction to a particular domain (e.g., “I appreciate my body for what it is capable of doing” and “I am grateful that my body enables me to engage in activities that I enjoy or find important”) [80]. The unidimensional structure of the scale was validated in the Arabic language among the Lebanese population [81] (α = 0.95).

*The Eating Attitudes Test-7 (EAT-7):* The EAT-7, validated in the Arabic language in Lebanon as a one-factor structure [82], is a shortened 7-item version of the original EAT-26 [83]. It is used to assess disordered eating attitudes (e.g., “Avoid eating when I am hungry”). Response options range between “infrequently/almost never/ never” (scored as 0) to “always” (scored as 3). The total score range between from 0 and 21, calculated as the sum of all items. Higher sums indicate greater disordered eating attitudes [82, 83] (α = 0.90).

### Study 3: pregnant women

#### Study design and procedures

In June and July 2021, we conducted a cross-sectional study targeting Lebanese pregnant women aged 18 years or older. Due to the COVID-19 pandemic and the restrictions on face-to-face interviews, we employed the snowball sampling method to adhere to governmental directives. To ensure a wide reach, we created a questionnaire using Google Forms, which included standardized measures. The survey link was then shared through social media platforms such as Facebook and WhatsApp, targeting participants residing in the five districts of Lebanon (Beirut, Mount Lebanon, North, South, and Bekaa). Additionally, women were encouraged to share the link with other pregnant individuals. By adopting a confidential self-administered approach, we aimed to protect privacy, minimize social influences, and maintain the integrity of participants' responses. In total, 433 pregnant women were reached.

#### Questionnaires and measures

The distributed questionnaire was divided into two sections. The first section was the same as studies 1 and 2. The second section included the following scales:

*The Body Dissatisfaction Scale (BDS) of the Eating Disorder Inventory* [17]: The description is given in study 1.

*The Restraint Scale of the Dutch Eating Behavior Questionnaire (DEBQ-R):* The description is provided in study 1. In the pregnant women population, α = 0.93.The description is provided in study 1. In the pregnant women population, α = 0.85.

*The Arabic version of the Disordered Eating Attitudes in Pregnancy Scale (A-DEAPS):* The A-DEAPS is a concise and effective screening tool using Yes-or-No questions. It contains ten questions assessing disordered eating attitudes in pregnancy, such as “I have wanted my pregnancy body to be small, like I am “just bump” (i.e., only my stomach appears to have grown, with no weight or shape changes to other areas of my body)” and “I have attempted to stop the changes occurring to my body during pregnancy”. Bannatyne et al. generated the original DEAPS with the intention of creating a trustworthy tool for screening for disordered eating symptoms unique to pregnancy [84]. The A-DEAPS scores vary between 0 and 10, and higher scores represent increased disordered eating attitudes during pregnancy. The Arabic items converged on one factor solution; the validated version of this scale was used [85] (α = 0.81).

*The Lebanese Anxiety Scale (LAS-10):* The LAS-10 is a short item designed to assess anxiety symptoms among the Lebanese population, including the following items “I have an anxious mood (worries, anticipation of the worst, fearful anticipation, irritability)” and “I feel that difficulties are piling up so that I cannot overcome them”. The higher the score, the more intense the anxiety. Items of the scale converged on one item [86] (α = 0.90).

### Statistical analysis

#### Confirmatory factor analysis (CFA)

There were no missing responses in the dataset. We used data from the second split-half in the adult sample to conduct a CFA using the SPSS AMOS v.29 software. Our intention was to test the BDS model obtained from the EFA results on the first split-half subsample. Evidence of convergent validity was assessed in this subsample in adults and the total sample in adolescents and pregnant women using the average variance extracted (AVE) values of ≥ 0.50 considered adequate [87].

#### Measurement invariance

To examine sex and BMI invariance of BDS scores, we conducted multi-group CFA [88] using the total samples in adults and adolescents. The BMI variable was dichotomized in two categories (≤ 25 and > 25 kg/m^2^ in adults and zBMI status (i.e., low-to-average weight [zBMI <  + 1SD] vs. higher weight [zBMI >  + 1SD] in adolescents). Measurement invariance was assessed at the configural, metric, and scalar levels [89]. Configural invariance implies that the latent BDS variable(s) and the pattern of loadings of the latent variable(s) on indicators are similar across sexes (i.e., the unconstrained latent model should fit the data well in both groups). Metric invariance implies that the magnitude of the loadings is similar across sexes; this is tested by comparing two nested models consisting of a baseline model and an invariance model. Scalar invariance implies that both the item loadings and item intercepts are similar across sexes and is examined using the same nested-model comparison strategy as with metric invariance. Lastly, strict invariance implies that residual invariance is equal across groups [88]. We accepted ΔCFI ≤ 0.010 and ΔRMSEA ≤ 0.015 or ΔSRMR ≤ 0.010 as evidence of invariance [88, 90]. We aimed to test for sex differences on latent BDS scores using an independent-samples *t*-test only if scalar or partial scalar invariance were established.

#### Further analyses

Reliability analysis in all samples was assessed using McDonald’s ω and Cronbach’s α, with values greater than 0.70 reflecting adequate reliability [91, 92]. The total BDS scores followed a normal distribution, with skewness and kurtosis values varying between − 1 and + 1 [93]. To assess convergent and divergent validity, we examined bivariate correlations between total BDS scores, and the additional measures included in the survey using the Pearson test. Student *t* test was used to compare two means. Based on Cohen [94], values ≤ 0.10 were considered weak, ~ 0.30 were considered moderate, and ~ 0.50 were considered strong correlations.

## Results

### Participants

The sociodemographic characteristics of the participants are summarized in Table 1.

**Table 1:** Sociodemographic characteristics of the participants in the three populations

	Adolescents	Adults	Pregnant women
Sex			
Male	135 (24.3%)	348 (42.1%)	–
Female	420 (75.7%)	478 (57.9%)	433 (100%)
Age (years)	16.66 ± 1.00 [min = 15; max = 18]	25.42 ± 8.44 [min = 18; max = 75]	28.55 ± 4.63 [min = 19; max = 43]
Household crowding index (persons/room)	0.99 ± 0.52	1.42 ± 0.88	0.82 ± 0.44
Body mass index (kg/m^2^)	22.38 ± 4.00	23.81 ± 4.83	27.54 ± 14.05
Pregnancy trimester			
First			75 (17.4%)
Second			176 (40.8%)
Third			180 (41.8%)

#### Confirmatory factor analysis of different models

A CFA was conducted on each total sample. In adolescents, we applied a correlation between residuals of items 1–3 and 8-F1 (Table 2, Model 2a). In adults; because of high modification indices, we added a correlation between residuals of items 1–2 and 3–4 (Table 2, Model 2b), whereas in pregnant women, we applied correlations between items 1–2 and 2-Factor 2 (Table 2, Model 2c). Consequently, the fit indices improved greatly. The standardized estimates of factor loadings were all adequate (see Table 3). The convergent validity for this model was satisfactory, with the AVE values exceeding 0.5 in all samples. Finally, the McDonald’s omega values were adequate in all samples.

**Table 2:** Fit indices of the confirmatory factor analyses conducted on the total sample among adolescents, adults, and pregnant women

	χ^2^(df)	CFI	TLI	RMSEA	90% CI	SRMR
Model 1: One-factor solution						
Model 1a	684.16/27 = 25.34	0.673	0.563	0.210	0.196, 0.223	0.016
Model 1b	1664.49/27 = 61.65	0.541	0.388	0.271	0.260, 0.282	0.020
Model 1c	630.60/27 = 23.36	0.609	0.479	0.227	0.212, 0.243	0.016
Model 2: Two-factor solution						
Model 2a	96.15/24 = 4.01	0.964	0.946	0.074	0.059, 0.089	0.032
Model 2b	103.21/24 = 4.30	0.978	0.967	0.063	0.051, 0.076	0.044
Model 2c	91.08/24 = 3.80	0.957	0.935	0.080	0.063, 0.098	0.049

Model 1 refers to all BDS items loading on one factor; Model 2 refers to BDS items loading on two factors (Factor 1: positively-worded and Factor 2: negatively-worded items). Model 1a and 2a were conducted in adolescents; Models 1b and 2b were conducted in adults; Models 1c and 2c were conducted in pregnant women. Model 2a: correlations added between items 1–6 and 8-Factor 1. Model 2b: correlations added between residuals of items 1–2 and 3–4. Model 2c: correlations added between items 3–4 and 4-Factor 2

**Table 3:** Factor loadings derived from the exploratory factor analyses (EFA) and standardized estimates of factor loadings from the confirmatory factor analysis (CFA) in all groups

BDS items	CFA in adolescents	CFA ion subsample 2 of adults	CFA in pregnant women
1-I think that my stomach is too big	0.68	0.67	0.47
2-I think that my thighs are too large	0.79	0.74	0.83
3-I think that my stomach is just the right size	0.68	0.59	0.51
4-I feel satisfied with the shape of my body	0.80	0.70	0.61
5-I like the shape of my buttocks	0.67	0.73	0.61
6-I think my hips are too big	0.70	0.84	0.79
7-I think that my thighs are just the right size	0.81	0.80	0.85
8-I think my buttocks are too large	0.57	0.80	0.71
9-I think that my hips are just the right size	0.81	0.83	0.88
AVE	0.53	0.56	0.50
McDonald’s ω	0.80	0.81	0.76
Cronbach’s α	0.81	0.82	0.81

Factor 1 = Body Satisfaction (Negatively-worded items: 1, 2, 6, 8); Factor 2 = Body Dissatisfaction (Positively-worded items: 3, 4, 5, 7, 9)

### Sex invariance

Indices in Table 4 indicate that configural, metric, scalar and partial strict invariance was supported across sexes in the adolescent and adult samples (Table 4). Among adolescents, a significantly higher mean BDS score was found in females (*M* = 12.81, *SD* = 6.60) compared to males (*M* = 11.00, *SD* = 5.65), *t*(553) = 2.866, *p* = 0.004, *d* = 0.294. Among adults, a significantly higher mean BDS score was found in males (*M* = 12.49, *SD* = 4.49) compared to females (*M* = 11.60, *SD* = 5.32), *t*(824) = 2.531, *p* = 0.012, *d* = 0.180.

**Table 4:** Measurement Invariance across Sexes

Model	CFI	RMSEA	SRMR	Model Comparison	ΔCFI	ΔRMSEA	ΔSRMR
Model 1: Adolescents							
Configural	0.947	0.065	0.053				
Metric	0.929	0.070	0.071	Configural vs. metric	0.018	0.005	0.018
Scalar	0.918	0.071	0.075	Metric vs. scalar	0.011	0.001	0.004
Strict	0.901	0.073	0.092	Scalar vs. strict	0.017	0.002	0.017
Model 2: Adults							
Configural	0.968	0.055	0.027				
Metric	0.962	0.056	0.029	Configural vs. metric	0.006	0.001	0.002
Scalar	0.959	0.055	0.029	Metric vs. scalar	0.003	0.001	< 0.001
Strict	0.944	0.060	0.050	Scalar vs. strict	0.015	0.005	0.021

*CFI* Comparative fit index; *RMSEA* Steiger-Lind root mean square error of approximation; *SRMR* Standardised root mean square residual

Furthermore, the results in Table 5 indicate that configural, metric, scalar and strict invariance was supported across BMI in the adult sample. A significantly higher body dissatisfaction score was found in overweight/obese participants compared to those with a normal BMI (*M* = 13.74, *SD* = 4.65 vs. *M* = 11.07, *SD* = 4.94; *t*(824) = − 7.525, *p* < 0.001).

**Table 5:** Measurement invariance across body mass index

Model	CFI	RMSEA	SRMR	Model Comparison	ΔCFI	ΔRMSEA	ΔSRMR
Model: Adults (BMI ≤ *25 and* > *25)*							
Configural	0.978	0.044	0.034				
Metric	0.976	0.043	0.034	Configural vs. metric	0.002	0.001	< 0.001
Scalar	0.968	0.047	0.034	Metric vs. scalar	0.008	0.004	< 0.001
Strict	0.961	0.049	0.036	Scalar vs. strict	0.007	0.002	0.002

*CFI* Comparative fit index; *RMSEA* Steiger-Lind root mean square error of approximation; *SRMR* Standardised root mean square residual. Measurement invariance across BMI could not be done in adolescents since all participants had a zBMI ≤ 1 SD

### Convergent and divergent validity

In adolescents, higher body dissatisfaction was significantly associated with greater depression, anxiety, restrained eating, and orthorexia nervosa tendencies (higher DOS scores).

In the adult sample, higher body dissatisfaction was significantly associated with lower body appreciation and lower functionality appreciation as well as higher EAT-7 scores (more disordered/inappropriate eating), indicating convergent validity.

Among pregnant women, higher body dissatisfaction was significantly associated with greater depression, anxiety, restrained eating, and disordered eating attitudes during pregnancy.

Higher BMI was significantly associated with higher body dissatisfaction in adults and adolescents, but not pregnant women (Table 6).

**Table 6:** Correlation matrix of the scales with the body dissatisfaction subscale scores in all groups

	Adolescents	Adults	Pregnant women
Body appreciation		− 0.41***	
Functionality appreciation		− 0.28***	
Eating attitudes		0.14**	0.45***
Depression	0.33***		0.26***
Anxiety	0.17***		0.30***
Restrained eating	0.37***		0.35***
Orthorexia nervosa	0.11**		
Body MASS index	0.41***	0.25***	0.05ϒ

**p < 0.01; ***p < 0.001; ϒ refers to BMI during pregnancy

### Comparison of body dissatisfaction scores between adults and adolescents

No significant difference was found between adults (*M* = 11.98, *SD* = 5.01) and adolescents (*M* = 12.37, *SD* = 6.42), *t*(1379) = − 1.205, *p* = 0.228 in terms of body dissatisfaction scores, pointing to the pervasiveness of body dissatisfaction among age groups in our population.

## Discussion

Our findings supported the reliability, cross-sex invariance, factorial validity, and convergent/divergent validity of the BDS in our three study groups. The BDS was reliable, consistent with previous findings across multiple nations [18]. Our findings also showed that the bidimensional component structure of the BDS scores was the same for men and women in both our adult and adolescent populations, in line with a previous study conducted in the US [95]. This property (i.e., sex invariance) allows for meaningful comparisons of thinness-oriented body dissatisfaction between men and women using the BDS.

The two-factor structure yielded by the EFA results had adequate fit to the model in the three populations. Ultimately, scores of the negatively-worded items are reversed, so that the total score reflects and quantifies body dissatisfaction symptoms only. However, since no previous study has approached the factor structure of the BDS in this manner, there are no available reported findings for comparison to ours.

Consistently with our hypothesis, we did not need to eliminate item 1 (i.e., dissatisfied with the size of stomach) in the pregnant women population, in contrast to the Hungarian study [48]. This finding is however congruent with a Delphi consensus study on the symptoms of disordered eating during pregnancy, which highlighted the desire for a small stomach/abdomen size [96]. In point of fact, there is evidence that body dissatisfaction may persist, even escalate, during pregnancy [41, 97]. In addition, distinctly from the well-known types of eating disorder symptoms, a new phenomenon called “Pregorexia” has been increasingly yet sparsely studied over the past few years and identified as an atypical eating-related condition specifically linked to pregnancy [98]. Pregorexia alludes to the attempts deployed by pregnant women to circumvent the natural weight gain in pregnancy through diet and excessive exercise, in order to remain thin during pregnancy [98–100]. Women may then exhibit an intense desire for a small pregnant belly [96]. Since this condition has been described in Lebanon [45, 85], this peculiar symptom (i.e., thinking one’s stomach is too big) might have been relevant to some pregnant women in the Lebanese population. The observed incongruities in the BDS factor structure between pregnant women in Lebanon and Hungary thus shed light on the probable higher presence of pregorexia in some societies, including the Lebanese one, compared to others.

In terms of convergent and divergent validity, higher body dissatisfaction scores were significantly associated with greater psychopathology (i.e., depression and anxiety) as well as numerous eating disorder symptoms (i.e., bulimic and anorexic tendencies, restrained eating patterns, orthorexia nervosa tendencies, and pregorexia) in our study. These findings converge to the existing relationships in the literature [5, 41, 56, 59, 75, 76, 101]. For instance, body dissatisfaction had a unique effect on poor general psychological well-being among Hispanic college students of both sexes [5]. Moreover, body dissatisfaction, as measured by the BDS, has been significantly associated with eating disorders such as anorexia nervosa and bulimia nervosa in the Taiwanese population [22]. On the other hand, we found that body dissatisfaction was inversely correlated with body appreciation and functionality appreciation. Accordingly, prior research has shown that the focus on the body’s functionality over its look may significantly enhance body image [62, 63]. As such, appreciative body functionality is apt to bolster body appreciation and thus reduce negative body image [64].

Overall, all the aforementioned findings provided support for the reliability and construct validity of the Arabic version of the BDS. The results of our study, along with the similarities observed in comparison to international studies, lend support to the cross-cultural applicability of the BDS and its potential usefulness within the Lebanese population.

### Limitations and future directions

Despite being the first report providing a validated Arabic tool to assess body image dissatisfaction in Arabic-speaking adults, adolescents, and pregnant women, our study presents some limitations. The cross-sectional design restrained our ability to assess additional psychometric properties of the Arabic version of the BDS, principally test–retest. No scales assessing psychological symptoms among adults were used. Weight and height were self-reported by participants. A selection bias is present since all samples were recruited via the snowball sampling technique (convenience sampling), which hinders the generalizability of the findings. In addition, the results cannot be generalized to other groups, such as children, older adults, and patients with eating disorder symptoms. Further studies adopting longitudinal designs and in other Arabic-speaking countries are still warranted to confirm our findings.

## Conclusion

In sum, our findings demonstrated the validity and reliability of the Arabic version of the BDS for evaluating body image dissatisfaction in different community groups, highlighting its applicability among Arabic-speaking people of both sexes as well as during pregnancy. In light of our findings, we endorse the use of the BDS by healthcare professionals in Arabic-speaking countries in order to detect thinness-oriented body dissatisfaction in an appropriate and timely manner and ease early referral to a specialist, thereby preventing the deleterious health-related risks associated with this condition.

### Supplementary Information

**Additional file 1:** Body dissatisfaction subscale of the eating disorder inventory-second version EDI-2.

## Data Availability

All data generated or analyzed during this study are not publicly available due to the restrictions by the ethics committee. The dataset supporting the conclusions is available upon request to the corresponding author.

## Abbreviations

BDS: Body dissatisfaction subscale
EDI: Eating disorder inventory
BAS-2: Body appreciation scale-2
FAS: Functionality appreciation scale
EAT-7: Eating attitudes test-7
PHQ-9: Patient health questionnaire
LAS: Lebanese anxiety scale
DEBQ-R: Restraint scale of the dutch eating behavior questionnaire
DOS: Düsseldorf orthorexia scale
A-DEAPS: Disordered eating attitudes in pregnancy scale
EFA: Exploratory factor analysis
KMO: Kaiser–Meyer–Olkin
PA: Parallel analysis
WRMSR: Weighted root mean square residual
CFA: Confirmatory factor analysis
AVE: Average variance extracted
US: United States
