# Supplementary material for: Validation of an Arabic version of the eating disorder inventory’s body dissatisfaction subscale among adolescents, adults, and pregnant women
Source: J Eat Disord. 2023 Oct 19;11:187. doi: 10.1186/s40337-023-00911-y (PMC10588257; doi:10.1186/s40337-023-00911-y)
Supplement: Supplementary file 1 — Additional file 1: Body dissatisfaction subscale of the eating disorder inventory-second version EDI-2. [file 40337_2023_911_MOESM1_ESM.docx]

**Body dissatisfaction subscale of the eating disorder inventory-second version EDI-2**

|  | أبدًا | نادرًا | في بعض الأحيان | في كثير من الأحيان | بشكل عام | دائمًا |
| --- | --- | --- | --- | --- | --- | --- |
| 1. أجد بطني كبير جدًا. |  |  |  |  |  |  |
| 1. أجد أردافي كبيرة جدًّا. |  |  |  |  |  |  |
| 1. أعتقد أنّ حجم بطني مناسب لجسمي. |  |  |  |  |  |  |
| 1. إنّني راضٍ عن شكل جسدي. |  |  |  |  |  |  |
| 1. أنا أحب شكل مؤخّرتي. |  |  |  |  |  |  |
| 1. أعتقد أنّ أوراكي كبيرة جدًا. |  |  |  |  |  |  |
| 1. أعتقد أنّ حجم فخذي مناسب لجسمي. |  |  |  |  |  |  |
| 1. أجد مؤخّرتي كبيرة جدًا. |  |  |  |  |  |  |
| 1. أعتقد أنّ حجم أوراكي مناسب لجسمي. |  |  |  |  |  |  |
